# Sortilin is associated with progranulin deficiency and autism‐like behaviors in valproic acid‐induced autism rats

**DOI:** 10.1111/cns.70015

**Published:** 2024-09-01

**Authors:** Ailing Liao, Wenxia Zheng, Shali Wang, Nashi Wang, Yingbo Li, Di Chen, Yan Wang

**Affiliations:** ^1^ NHC Key Laboratory of Birth Defects and Reproductive Health Chongqing Population and Family Planning Science and Technology Research Institute Chongqing China; ^2^ Institute of Neuroscience, School of Basic Medical Science Chongqing Medical University Chongqing China; ^3^ Library/Archive Chongqing Medical University Chongqing China

**Keywords:** autism spectrum disorder, dendrites, inflammation, NF‐κB pathway, PGRN, *SORT1*

## Abstract

**Introduction:**

Neuroinflammation and microglial activation‐related dendritic injury contribute to the pathogenesis of Autism Spectrum Disorder (ASD). Previous studies show that Progranulin (PGRN) is a growth factor associated with inflammation and synaptic development, but the role of PGRN in autism and the mechanisms underlying changes in PGRN expression remain unclear.

**Aims:**

To investigate the impact of PGRN in autism, we stereotactically injected recombinant PGRN into the hippocampus of ASD model rats. Additionally, we explored the possibility that sortilin may be the factor behind the alterations in PGRN by utilizing *SORT1* knockdown. Ultimately, we aimed to identify potential targets for the treatment of autism.

**Results:**

PGRN could alleviate inflammatory responses, protect neuronal dendritic spines, and ameliorate autism‐like behaviors. Meanwhile, elevated expression of sortilin and decreased levels of PGRN were observed in both ASD patients and rats. Enhanced sortilin levels facilitated PGRN internalization into lysosomes. Notably, suppressing *SORT1* expression amplified PGRN levels, lessened microglial activation, and mitigated inflammation, thereby alleviating autism‐like behaviors.

**Conclusion:**

Collectively, our findings highlight elevated sortilin levels in ASD rat brains, exacerbating dendrite impairment by affecting PGRN expression. PGRN supplementation and *SORT1* knockdown hold potential as therapeutic strategies for ASD.

## Graphical abstract

In hippocampal neurons of ASD rats, elevated sortilin levels are associated with increased delivery of PGRN to lysosomes, where the endocytosed PGRN is rapidly degraded. This high level of sortilin, coupled with low PGRN expression, could result in the activation of microglia and the NF‐κB pathway. The activation of the NF‐κB pathway might further promote microglial activation. Collectively, these factors contribute to synaptic loss and a reduction in dendritic spines in the hippocampus, leading to autism‐like behaviors.

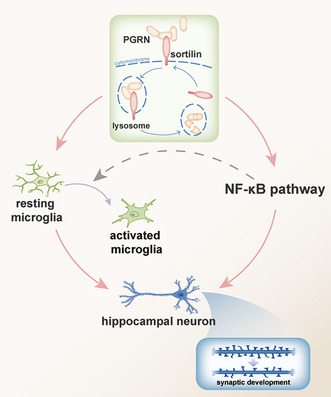

## 1 INTRODUCTION

Autism spectrum disorders (ASD) encompasses a group of neurodevelopmental disorders characterized by impaired social communication and restricted interests or repetitive behaviors. Despite the identification of numerous risk factors associated with ASD, effective therapeutic interventions for the disorder remain elusive. Currently, behavioral intervention is the predominant approach used in ASD.^1^ The etiology of ASD is multifactorial and involves genetic mutations,^2^ imbalance between excitatory and inhibitory factors, and inflammation.^3^, ^4^ Studies have consistently reported increased microglial density in the brains of individuals with ASD.^5^, ^6^ The symptoms of ASD can be alleviated by controlling microglial activation and reducing inflammation,^7^, ^8^ indicating that inflammation and microglial activation are potential therapeutic targets for ASD. Existing studies on the neurobiological basis of ASD propose the involvement of the nuclear factor kappa B (NF‐κB) pathway in the etiology of ASD.^9^, ^10^ Additionally, NF‐κB is abundantly expressed in the microglia, and NF‐κB activation is often accompanied by significant microglial activation and neuroinflammation.^11^ IKKβ and p65, key components of the NF‐κB signaling pathway, play a pivotal role in this process. Elevated levels of phosphorylated IKKβ and p65 indicate the activation of the NF‐κB pathway.^12^, ^13^ Moreover, microglia are closely related to synaptic pruning, as they can remove synapses through phagocytosis and interact with terminal boutons and dendritic spines.^14^ Sellgren proposed that abnormal synaptic pruning caused by microglial overactivation can lead to the development of schizophrenia.^15^

Progranulin (PGRN), a secretory growth factor widely recognized for its neuroprotective properties in neurodegenerative diseases, has been implicated in various cellular processes, including cell proliferation, regulation of inflammation, and synaptic development.^16^ In the central nervous system, PGRN is expressed in microglia and neurons.^17^ Numerous studies have demonstrated the substantial anti‐inflammatory and neuroprotective properties of PGRN. Furthermore, PGRN promotes neurite outgrowth and neuronal differentiation,^18^ as well as reduces axonal injury through the downregulation of TNF‐α expression in traumatic brain injury.^19^ Similarly, Tang demonstrated that PGRN can directly bind to TNFR, acting as a competitive antagonist to inhibit the TNF‐α/NF‐κB pathway.^20^ However, the potential anti‐inflammatory effects of PGRN and the factors that influence PGRN expression in ASD are unclear.

Sortilin, a versatile receptor protein encoded by the *SORT1* gene, is involved in cellular transportation. In the central nervous system, sortilin is involved in neuroinflammation and brain damage.^21^ Notably, recent studies have reported elevated serum levels of sortilin in children with ASD.^22^, ^23^ Several studies have indicated that sortilin serves as a high‐affinity trafficking receptor, regulating the concentration of PGRN by mediating its endocytosis and clearance. Sortilin binds to the C‐terminal of PGRN, facilitating its rapid transport to the lysosome for degradation and subsequent return to the cell surface.^24^, ^25^ Sortilin downregulation reduces PGRN clearance and increases the extracellular PGRN concentration, leading to a five‐fold increase in the serum PGRN levels in mice, making *SORT1* a potential target for increasing the PGRN levels in patients with FTLD.^16^, ^26^, ^27^ However, whether sortilin can regulate PGRN expression in microglia and impact microglial activation and inflammatory reactions in ASD remains unknown.

In this study, we aimed to examine the role of PGRN in ASD and investigate whether sortilin regulates PGRN, thereby subsequently affecting microglial activation and inflammatory responses in ASD through its involvement in lysosomal delivery.

## 2 MATERIALS AND METHODS

### 2.1 Study participants

Fasting blood samples were collected from 11 to 12 normal children and 13 children with ASD at Chongqing Maternal and Child Health Hospital. The children with ASD included 9 males and 4 females (mean age ± SD = 5.7 ± 2.9 years), diagnosed based on the Diagnostic and Statistical Manual of Mental Disorders, 5th edition.^28^ The normal children included 7 males and 5 females (mean age ± SD = 5.8 ± 1.5 years). These healthy children demonstrated no clinical findings suggestive of neuropsychiatric disorders. None of the participants had a recent history of infection or fever.

The study protocol was approved by the Medical Research Ethics Committee of Chongqing Medical University (IACUC‐CQMU‐2023‐0334). Written informed consent was obtained from all parents in accordance with the principles of the Declaration of Helsinki (2008).

### 2.2 Enzyme‐linked immunosorbent assay (ELISA)

Blood samples were collected in vacutainer tubes, held at 4°C for 30 min, and then centrifuged at 3500 rpm for 5 min to obtain serum samples. Human Quantikine ELISA Kits (manufactured by Wuhan ColorfulGene Biological Technology, China) were employed to detect serum levels of Sortilin and Tumor Necrosis Factor Alpha (TNF‐α) following the manufacturer's protocol. PGRN levels in human serum and TNF‐α levels in rat hippocampal tissue were measured using ELISA kits (produced by Boster Biological Technology, Wuhan, China).

### 2.3 Animal model

Animal procedures were evaluated and approved by the Ethics Committee of Experimental Animals of Chongqing Medical University and followed the Guide for the Care and Use of Laboratory Animals (NIH Publication No.85‐23, revised 1996). Timed‐pregnant Sprague–Dawley rats (Experimental Animal Center in Chongqing Medical University, Chongqing, China) were randomly assigned on gestational day (GD) 12.5 to receive intraperitoneal injections of either sterile saline or 600 mg/kg Sodium Valproate (dissolved in sterile saline). Male offspring of these rats were selected as the experimental subjects. The male offspring from dams receiving sterile saline were assigned to the Sham group, while the male offspring from dams receiving VPA were randomly divided into the VPA + vehicle group, VPA + PGRN group, VPA + Si‐SORT1 group, and VPA + NC group for subsequent experiments.

### 2.4 Stereotaxic surgery

On PND10, all rats were administered inhalation anesthesia with 2.5% isoflurane and secured onto a stereotaxic apparatus (RWD Life Technology, Shenzhen, China). A microlitre syringe was inserted into each side of hippocampus using the following coordinates: 2.3–2.5 mm posterior, 2.5–2.8 mm lateral, 3–3.5 mm deep (relative to bregma). Rats were treated with the different 5 μL drug injections: 0.9% sterile saline (VPA + vehicle group), 50 ng/mL recombinant PGRN (r‐PGRN) (SinoBiological, Beijing, China) dissolved in 0.9% sterile saline (VPA + PGRN group), SORT1 knockdown lentivirus (VPA + Si‐SORT1 group), and scramble lentivirus (non‐targeting sequences) (VPA + NC group). Rats of the Sham group underwent the same surgical procedure but did not receive any drug injections. On PND 17, the VPA + PGRN group was injected with rPGRN once again, using the same dosage as previously. Meanwhile, those in the Sham group and the VPA + vehicle group who matched with the VPA + PGRN group also received the same treatment as before. Criteria for postoperative analgesic treatment were predefined and included signs such as aggression, piloerection, hunched posture, or rapid and shallow breathing, in accordance with national and international guidelines.^29^ Post‐surgery, each rat was monitored for indicators like body weight loss from baseline, wound healing, and behavioral changes, porphyrin staining (a sign of stress, illness, or poor environmental conditions for rats). We employed a comprehensive animal welfare scoring table, adapted from Hampshire and co‐workers,^30^ to ensure objective and systematic assessment. These evaluations combined various parameters to identify welfare impairments and ascertain if humane endpoints were needed. If signs indicated insufficient pain relief or if a combined score exceeded 0.4, euthanasia would be carried out. In this study, no animals reached this threshold.

### 2.5 Gene knock‐down

Purified plasmid DNA based on the pGLV1(U6/GFP) lentiviral vector drives a short hairpin DNA (shDNA) sequence (GCCCTGGAATTATGGAGAACA), was precisely engineered to target and silence the *SORT1* gene in rats. The correct construction and insertion of the shDNA sequence were rigorously confirmed through sequencing methods.

### 2.6 Behavioral analysis

#### 2.6.1 Three‐chamber social test

A transparent plastic chamber (120 × 45 × 40 cm) was divided into three equal‐sized compartments with removable walls. The test environment had controlled lighting, low noise levels, and appropriate temperature. Chambers were labeled A, central, and B, with side chambers (A and B) containing small wire cages. To capture the experiment, a camera was installed outside of the apparatus. Initially, the test rat was acclimated to the central chamber for 10 min. During the sociability test stage, an unfamiliar male rat of similar age (stranger A) was introduced into the cage in chamber A, while chamber B remained empty and served as the object. The camera recorded the test session for a duration of 10 min. In the social novelty preference test stage, another unfamiliar male rat of similar age (stranger B) was added to the cage in chamber B. The camera recorded another 10‐min session. The apparatus was cleaned with 75% ethanol between tests to minimize residual odors.

#### 2.6.2 Open field test and self‐grooming test

The apparatus consisted of a transparent square cage (100 × 100 × 40 cm) with the floor divided into 25 equal grids. Among these grids, 16 located adjacent to the walls were designated as circumambient grids, while the remaining nine grids were classified as central grids. Before testing, rats were habituated to the cage for 10 min. They were then placed in the central grid and allowed to explore the apparatus for an additional 10 min. During the test, a camera recorded several behavioral parameters, including the number of grids crossed, frequency of standing and climbing the walls, duration of self‐grooming, and number of excretions. The apparatus was cleaned with 75% alcohol between tests to minimize residual odors.

#### 2.6.3 Juvenile social play test

The juvenile social play test was conducted in an apparatus evenly paved with clean sawdust. An age‐matched, unfamiliar male rat was chosen as a social partner for the test rats. The test rats were allowed to habituate to the entire cage for 10 min before being placed with the social partner into the apparatus simultaneously. The experiment recorded the time spent engaging in active social behaviors such as licking, grooming, touching, and sniffing the partner, digging in the sawdust, and attacking the partner over a 10‐min period. The sawdust was thoroughly replaced between tests to minimize residual odors for subsequent tests.

### 2.7 Western blot

Hippocampal tissues were isolated on ice and mechanically lysed using ice‐cold RIPA buffer (Beyotime, Shanghai, China) supplemented with 1% (μL/μL) PMSF (Beyotime, Shanghai, China) and 1% (μL/μL) phosphatase inhibitor (Beyotime, Shanghai, China). After centrifugation at 12,000 rpm for 15 min at 4°C, the supernatants were collected. The protein concentration was quantified using the BCA assay (Beyotime, Shanghai, China). After adjusted to equal concentration, it was mixed with 5× SDS‐PAGE loading buffer and subjected to boiling for denaturation. Subsequently, 20 μg of protein per lane was separated on 8–12% SDS‐PAGE gel and transferred onto nitrocellulose membranes. Membranes were blocked with nonfat milk in TBST (Tris‐buffered saline containing Tween‐20) for 2 h and then incubated with primary antibodies overnight at 4°C. Primary antibodies used included anti‐β‐actin (1:100,000, ABclonal, China), anti‐Synaptophysin (1:320,000, ProteinTech, China), anti‐PSD95 (1:8000, CST, USA), anti‐TNF‐α (1:800, ABclonal, China), anti‐IL‐1β (1:1000, ABclonal, China), anti‐p‐IKKβ (1:1000, CST, USA), anti‐IKKβ (1:1000, CST, USA), anti‐p‐p65 (1:1000, Affinity, USA), anti‐p65 (1:1000, Affinity, USA), and anti‐sortilin (1:1000, Abcam, UK). After removing excess antibody solution, membranes were washed with TBST and incubated with either goat anti‐mouse IgG‐HRP (1:5000, Boster, China) or goat anti‐rabbit IgG‐HRP (1:5000, Boster, China) for 2 h at room temperature. Detection was performed using an ECL kit (Abcam, UK) according to the manufacturer's instructions after washing off unconjugated secondary antibodies with TBST. Densitometric analysis of bands was conducted using ImageJ software (version 1.53c).

### 2.8 Immunofluorescence in vivo

Rats were euthanized and perfused with PBS to remove residual blood. The brains were fixed in 4% paraformaldehyde (PFA) for 24 h and washed before dehydration with graded sucrose solutions (10%, 20%, and 30%). The brains were then sectioned into 10‐μm‐thick coronal slices using a frozen microtome (Leica, Germany). Sections were permeabilized with 0.5% Triton‐X in PBS for 30 min, blocked with 5% BSA for 1 h, and probed with primary antibodies NF‐κB‐p65 (1:500, Affinity, USA) and Iba1 (1:1000, Abcam, UK) overnight at 4°C. After washing, the sections were incubated with secondary antibodies Alexa Fluor®555 and Alexa Fluor®488 at room temperature for 1 h. Finally, sections were stained with DAPI (Sigma, USA). The morphology and colocalization of the sections were captured using a confocal microscope (Leica, Germany) and analyzed by ImageJ (version 1.53c). Morphological analysis and evaluation of microglia were carried out following methods described in relevant literature.^31^, ^32^

### 2.9 Golgi‐Cox staining

Golgi‐Cox staining is a specialized technique for visualizing neuronal dendrite and dendritic spine morphology. All procedures were performed according to the protocols of Golgi‐Cox OptimStain™ PreKit (Hitobiotch, USA). Brains were harvested as before, pretreated with the Kit and sliced into 100‐μm‐thick sections and dehydrated with different concentrations of alcohol. After stained, they were cleared in Xylene and covered up with neutral balsam. Dendritic spines of hippocampal neurons were observed and imaged using an Eclipse E100 microscope (Nikon, Japanese). For brain tissue, we employed a continuous sectioning method and sampled at consistent thickness intervals. From each rat, we used 3 slices, and for each slice, we selected 5 secondary dendrites, each with a length of 20–50 μm, for spine analysis using ImageJ software (version 1.53c).

### 2.10 Cell culture and drug treatment

BV2 microglia were cultured in sterile high‐glucose DMEM containing 100 mL/L fetal bovine serum, 100 IU/mL penicillin, and 100 μg/mL streptomycin at 37°C and 50 mL/L CO2. Two days after cell passage, cells were divided into four groups: CON, VPA, VPA + Si‐SORT1, and VPA + NC. The VPA + Si‐SORT1 group and VPA + NC group were transfected with Si‐SORT1 knockdown lentivirus and scramble lentivirus (non‐targeting sequences), respectively, for 3 days. Subsequently, all groups except the CON group were treated with 2.5 mmol/L VPA, while the CON group was treated with an equivalent volume of PBS.

### 2.11 Immunofluorescence in vitro

Following the treatment described above, the cells were washed with pre‐cooled PBS and fixed in pre‐cooled 4% PFA for 30 min. The cells were then washed three times and blocked in 5% BSA for 1 h. Subsequently, the cells were probed with primary antibodies anti‐PGRN (1:100, CST, USA) and anti‐LAMP1 (1:100, Abcam, UK) overnight at 4°C, followed by incubation with goat anti‐mouse Alexa Fluor®555 (1:100, Abcam, UK) and goat anti‐rabbit Alexa Fluor®488 (1:100, Beyotime Biotechnology, China) secondary antibodies for 1 h at room temperature. The cells were then stained with DAPI and sealed with nail polish. Laser scanning confocal microscopy and ImageJ software mentioned before were used to analyze the colocalization of anti‐PGRN and anti‐LAMP1.

### 2.12 Statistical analysis

Statistical analysis was conducted using GraphPad Prism software (version 9.0.0). Data are expressed as mean ± SEM. Prior to selecting the appropriate statistical test, the assumptions of normality and homogeneity of variance were assessed. All data underwent normal distribution testing prior to analysis. Specifically, when the sample size was less than 50, we employed the Shapiro–Wilk test to assess normality. For sample sizes equal to or greater than 50, the D'Agostino‐Pearson test was used to evaluate conformity to a normal distribution. For pairwise comparisons, an unpaired *t*‐test was employed if the data conformed to normal distribution and satisfied the homogeneity of variance test; otherwise, the Mann–Whitney *U* test was utilized. When variance was not homogeneous, Welch's correction was applied. In the case of multiple group comparisons, one‐way analysis of variance (ANOVA) with Sidak post hoc test was performed if the data followed a normal distribution. If the data failed to meet the homogeneity of variance test or normal distribution assumption, the Kruskal–Wallis test was implemented. A *p*‐value less than 0.05 was deemed statistically significant.

## 3 RESULTS

### 3.1 PGRN alleviated autism‐like behaviors in VPA‐induced ASD rats

**FIGURE 1:**
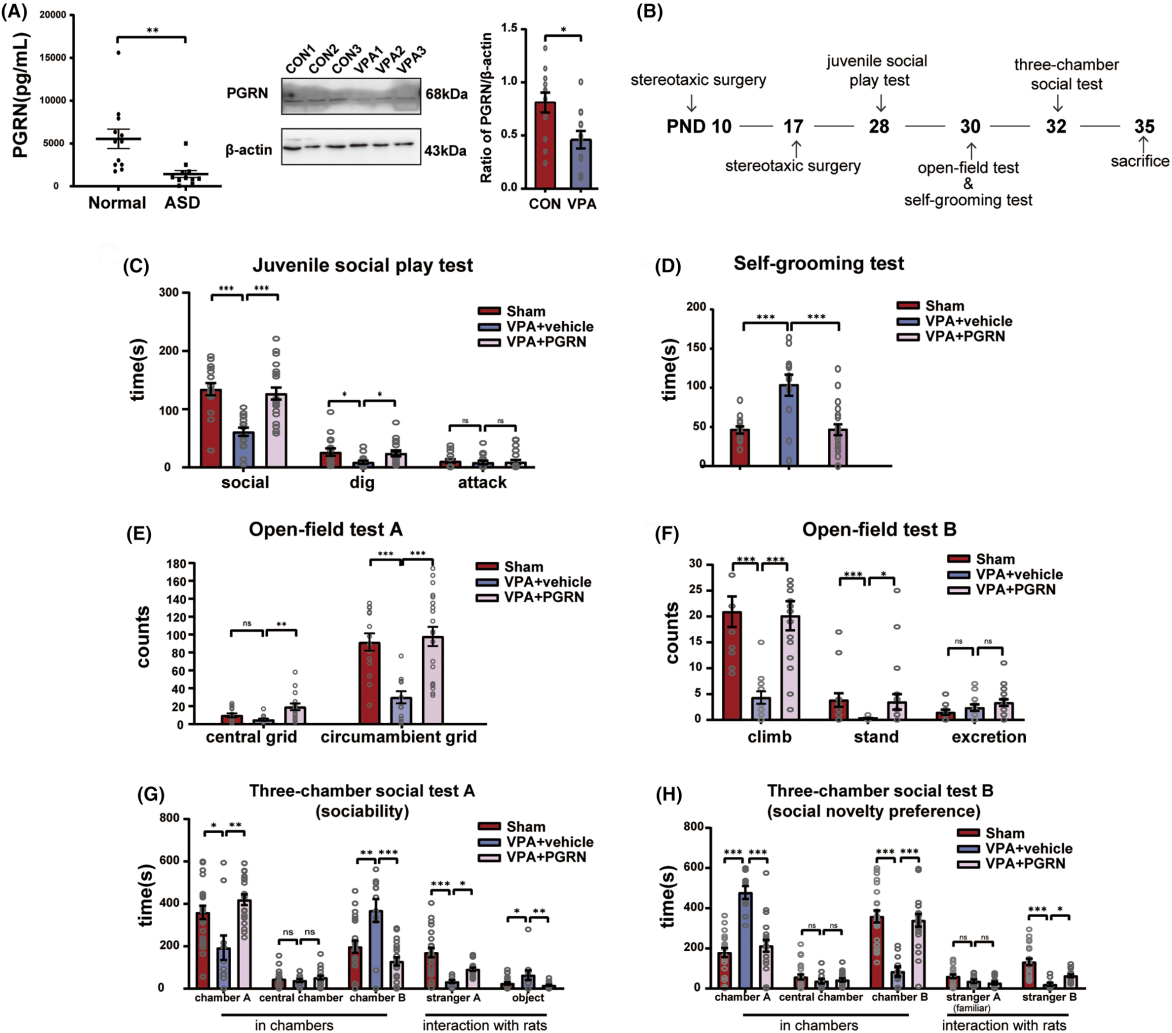
PGRN content in the hippocampus of VPA‐induced ASD rats and the impact of PGRN supplementation on autism‐like behaviors. (A) The results of the ELISA measurement of PGRN levels in the serum of children with autism and the Western blot measurement of PGRN levels in the hippocampus of VPA‐induced ASD rats at PND35. Sample size (*n*) of normal children: *n* = 12. Sample size (*n*) of ASD children: *n* = 13. Sample size (*n*) of rats: *n* = 5/group. The analysis was repeated four times, using an unpaired *t* test for statistical analysis. (B) Timeline of the stereotaxic surgery, autism‐related behavioral tests, and brain harvesting. (C) Results from the juvenile social play test showing the social time, digging time, and attack time. (D) Results from the self‐grooming test showing the grooming time. (E and F) Results from the open‐field test showing the central grid, circumambient grid, climb, stand, and excretion. (G and H) Results from the three‐chamber social test showing the time spent in the central, A, and B chambers, and interaction with strangers A and B. Sample size of rats per group (*n*): *n* = 13–14 (Sham), *n* = 11–12 (VPA + vehicle), and *n* = 19–20 (VPA + PGRN). All data are presented as mean ± SEM. One‐way ANOVA followed by Sidak post hoc test or Kruskal–Wallis test performed for multiple comparisons (ns: no significant difference, *p < 0.05, **p < 0.01, ***p < 0.001).

We initially examined the PGRN expression in the serum of ASD children using ELISA, and the PGRN expression in the hippocampus of VPA‐induced ASD rats using western blot analysis (Figure 1A). The results revealed a significant reduction in the PGRN expression in the serum of ASD children (*t* = 3.229, *df* = 21, *p* = 0.0034 for ASD vs. Normal) and in the hippocampus of the VPA group at postnatal day (PND) 35 (*t* = 2.816, *df* = 22, *p* = 0.01 for VPA vs. CON). ASD rats were treated with recombinant PGRN twice, and behavioral tests were used to observe the changes in autism‐like behaviors. The timeline of the surgical and behavioral tests is illustrated in Figure 1B.

The Juvenile Social Play test (Figure 1C) showed a notable increase in both social time (*F*_2,50_ = 16.11, *p* < 0.001; post hoc, *p* < 0.001 for VPA + vehicle vs. Sham, *p* < 0.001 for VPA + PGRN vs. VPA + vehicle) and dig time (*F*_2,50_ = 3.795, *p* = 0.03; post hoc, *p* = 0.03 for VPA + vehicle vs. Sham, *p* = 0.05 for VPA + PGRN vs. VPA + vehicle) with PGRN supplementation. The Self‐Grooming test (Figure 1D) demonstrated a significant reduction in the grooming duration with PGRN supplementation (*F*_2,43_ = 13.46, *p* < 0.001; post hoc, *p* < 0.001 for VPA + vehicle vs. Sham, *p* < 0.001 for VPA + PGRN vs. VPA + vehicle).

In the Open‐Field test (Figure 1E,F), the VPA + PGRN group exhibited an increase in the number of climbing events, standing events, and grid crossing (climb: Kruskal–Wallis statistic = 19.94, *p* < 0.001; post hoc, *p* < 0.001 for VPA + vehicle vs. Sham, *p* < 0.001 for VPA + PGRN vs. VPA + vehicle; stand: Kruskal–Wallis statistic = 13.11, *p* = 0.001; post hoc, *p* < 0.001 for VPA + vehicle vs. Sham, *p* = 0.05 for VPA + PGRN vs. VPA + vehicle; central grids: Kruskal‐Wallis statistic = 11.75, *p* = 0.003; post hoc, *p* = 0.35 for VPA + vehicle vs. Sham, *p* = 0.002 for VPA + PGRN vs. VPA + vehicle; circumambient grids: *F*_2,43_ = 12.21, *p* < 0.001; post hoc, *p* < 0.001 for VPA + vehicle vs. Sham, *p <* 0.001 for VPA + PGRN vs. VPA + vehicle).

In the sociability test stage of the three‐chamber social test (Figure 1G), the VPA + PGRN group demonstrated an increase in the duration spent in chamber A (*F*_2,40_ = 9.193, *p* = 0.0005; post hoc, *p* = 0.0012 for VPA + vehicle vs. Sham, *p* = 0.0007 for VPA + PGRN vs. VPA + vehicle) and a decrease in the time spent in chamber B (*F*_2,40_ = 12.51, *p* < 0.001; post hoc, *p* < 0.001 for VPA + vehicle vs. Sham, *p* < 0.001 for VPA + PGRN vs. VPA + vehicle). Concurrently, they exhibited an increased duration of interaction with stranger A (*F*_2,40_ = 11.33, *p* < 0.001; post hoc, *p* < 0.001 for VPA + vehicle vs. Sham, *p* = 0.007 for VPA + PGRN vs. VPA + vehicle) and a decreased interaction with the object (Kruskal–Wallis statistic = 11.77, *p* = 0.0028; post hoc, *p* = 0.0022 for VPA + vehicle vs. Sham, *p* = 0.0113 for VPA + PGRN vs. VPA + vehicle). In the social novelty preference stage (Figure 1H), the VPA + PGRN group exhibited reduced time spent in chamber A (*F*_2,40_ = 33.27, *p <* 0.001; post hoc, *p* < 0.001 for VPA + vehicle vs. Sham, *p* < 0.001 for VPA + PGRN vs. VPA + vehicle) and increased time spent in chamber B (*F*_2,40_ = 27.84, *p <* 0.001; post hoc, *p* < 0.001 for VPA + vehicle vs. Sham, *p* < 0.001 for VPA + PGRN vs. VPA + vehicle). Additionally, they showed a prolonged duration of interaction with stranger B (*F*_2,40_ = 11.29, *p <* 0.001; post hoc, *p* < 0.001 for VPA + vehicle vs. Sham, *p* = 0.01 for VPA + PGRN vs. VPA + vehicle).

These results suggest that PGRN alleviates social preference deficits, improves repetitive and rigid behavior, and enhances social competence in VPA‐induced ASD rats.

To determine the extent of surgical damage, we monitored the body weight, general appearance, porphyrin staining, gait and posture, appetite, and wound condition of the animals pre‐surgery and on postoperative days 1, 3, and 7. We found that the surgery did not cause significant trauma in the sham group, VPA + vehicle group, and VPA + PGRN group animals (see Table S1). Therefore, we believe that the behavioral changes observed are mainly influenced by the injection of PGRN, rather than by the surgery itself.

### 3.2 PGRN inhibits the NF‐κB pathway and microglial activation in the hippocampus of VPA‐induced ASD rats

**FIGURE 2:**
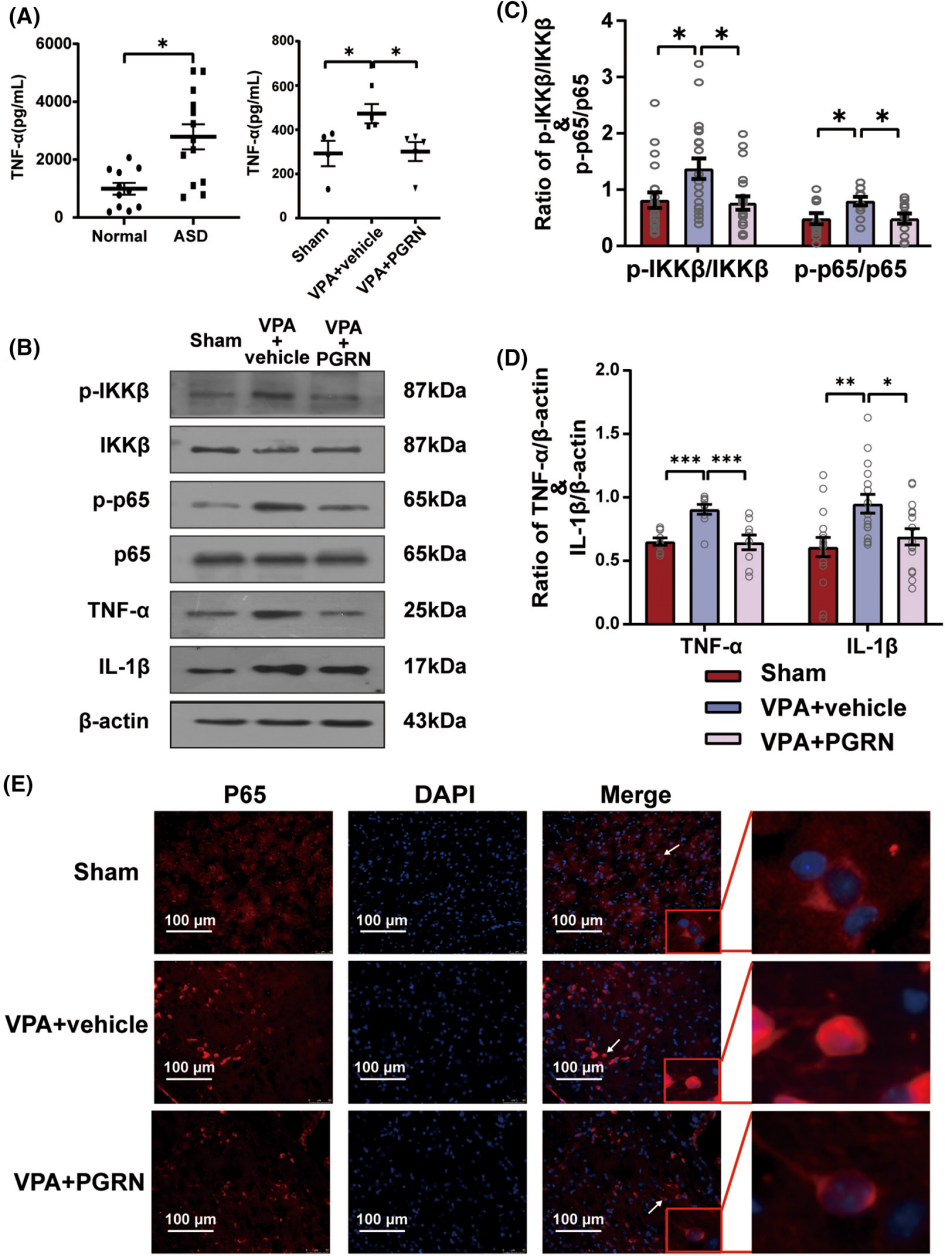
Inflammation in ASD, and the impact of PGRN supplementation on the NF‐kB pathway. (A) ELISA detection of TNF‐α levels in the serum of normal children and ASD children, as well as TNF‐α levels in the hippocampal tissue of three groups of rats. Sample size (*n*) of children: *n* = 11 (normal) and *n* = 13 (ASD), using unpaired *t* test used for statistical analysis. Sample size (*n*) of rats: *n* = 4 (Sham), *n* = 4 (VPA + vehicle), and *n* = 5 (VPA + PGRN), using one‐way ANOVA followed by Sidak post hoc test for statistical analysis. (B) Phosphorylation of IKKβ and p65; expression of TNF‐α and IL‐1β in the hippocampus of Sham, VPA + vehicle, and VPA + PGRN groups at PND35. (C) Statistical graph illustrating the ratio of p‐IKKβ to IKKβ and that of p‐p65 to p65 in the Sham, VPA + vehicle, and VPA + PGRN groups. Sample size (*n*): *n* = 5/group. The analysis was repeated 4–5 times, using one‐way ANOVA followed by Sidak post hoc test for statistical analysis. (D) Statistical graph illustrating the ratio of TNF‐α to β‐actin and that of IL‐1β to β‐actin. Sample size of rats (*n*): *n* = 5/group. The analysis was repeated 4–5 times, using one‐way ANOVA followed by Sidak post hoc test for statistical analysis. (E) Representative images showing subcellular localization of p65 in the hippocampus of Sham, VPA + vehicle, and VPA + PGRN groups. White arrow: Selected observation cell. Red box: Enlarged image of selected cells demonstrating the details. Original magnification: 400×. Scale bar: 100 μm. The images were obtained and edited using a confocal microscope. Data are presented as mean ± SEM (ns: no significant difference, *p < 0.05, **p < 0.01, ***p < 0.001).

We used enzyme‐linked immunosorbent assay (ELISA) to detect TNF‐α levels in the serum of normal and ASD children. The results revealed significantly elevated serum TNF‐α levels in ASD children (*t* = 3.534, *df* = 22, *p* = 0.002 for ASD vs. Normal), indicating an activation of the inflammatory response in ASD. At the same time, we also found higher expression levels of TNF‐α in the hippocampal tissue of the VPA + vehicle group compared to the Sham group, and PGRN could inhibit the increase of TNF‐α in the hippocampal tissue (*F*_2,10_ = 4.329, *p* = 0.0442; post hoc, *p* = 0.0499 for VPA + vehicle vs. Sham, *p* = 0.0489 for VPA + PGRN vs. VPA + vehicle) (Figure 2A). Subsequently, we used western blot analysis to assess the phosphorylation levels of IKKβ and p65 in the hippocampus of different groups of ASD rats at PND35 (Figure 2B–D). The results showed that PGRN supplementation significantly mitigated the increased levels of IKKβ and p65 phosphorylation in the hippocampus (IKKβ: *F*_2,57_ = 5.121, *p* = 0.009; post hoc, *p* = 0.02 for VPA + vehicle vs. Sham, *p* = 0.01 for VPA + PGRN vs. VPA + vehicle; p65: *F*_2,30_ = 4.172, *p* = 0.03; post hoc, *p* = 0.04 for VPA + vehicle vs. Sham, *p* = 0.04 for VPA + PGRN vs. VPA + vehicle). Moreover, the expression levels of the pro‐inflammatory cytokines TNF‐α and IL‐1β in the VPA + PGRN group exhibited a notable reduction compared to the VPA + vehicle group (*F*_2,24_ = 11.45, *p* < 0.001; post hoc, *p* < 0.001 for VPA + vehicle vs. Sham, *p* < 0.001 for VPA + PGRN vs. VPA + vehicle; *F*_2,45_ = 6.219, *p* = 0.004; post hoc, *p* = 0.003 for VPA + vehicle vs. Sham, *p* = 0.03 for VPA + PGRN vs. VPA + vehicle). Furthermore, we performed immunofluorescence staining to observe the subcellular localization of the p65 protein in the hippocampus of the different groups at PND35 (Figure 2E), which revealed reduced nuclear localization of the p65 protein in the VPA + PGRN group compared to the VPA + vehicle group. These results indicate that PGRN can reduce inflammation in the hippocampus.

**FIGURE 3:**
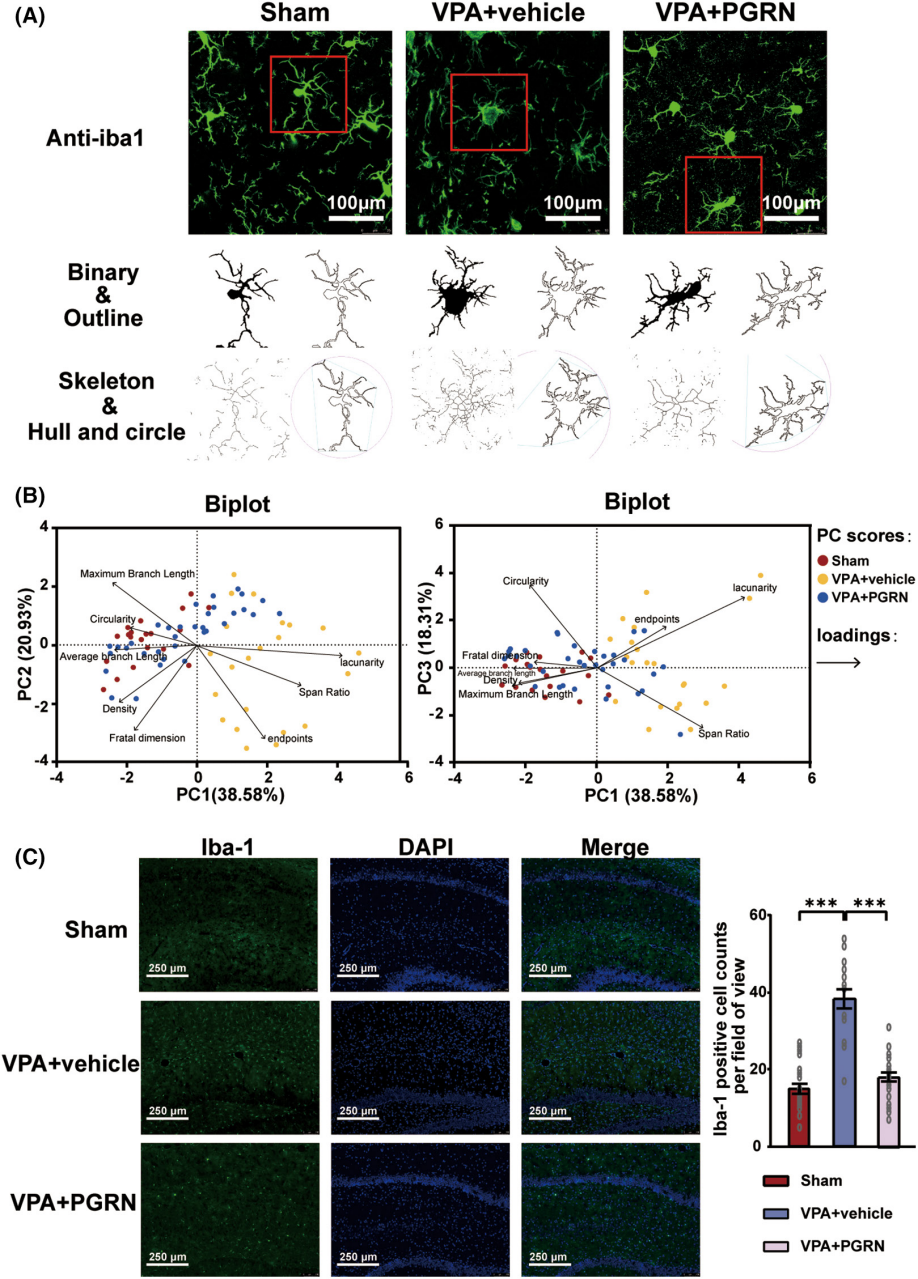
Impact of PGRN supplementation on microglial counts and morphological changes in the hippocampus. (A) Representative images of Iba1‐stained microglia morphology in the hippocampus of Sham, VPA + vehicle, and VPA + PGRN groups obtained using a confocal microscope. Original magnification: 630×. Scale bar: 100 μm. Cropped and analyzed photomicrographs (below) were acquired to obtain additional details. All analyses were performed using full‐sized photomicrographs. (B) Principal component analysis (PCA) of the morphological changes of microglia in the hippocampus of the Sham, VPA + vehicle, and VPA + PGRN group. The figure displays a biplot for PCA, analyzing eight morphological change indicators: Endpoints, average branch length, maximum branch length, density, span ratio, circularity, lacunarity, and fractal dimension. Percentages of variation for morphology of microglia are shown for the first three principal components (PC1, PC2, and PC3). In the biplot, each point represents the principal component (PC) score of a sample, and arrows represent loadings. The length of each arrow indicates the relative contribution of each indicator to the PCA, and the angles between arrows illustrate their correlations. Sample size of microglia per group (*n*): *n* = 20 (Sham), *n* = 23 (VPA + vehicle), and *n* = 35 (VPA + PGRN). (C) Representative images and quantification analysis of Iba1‐stained microglia counts in the hippocampus of the Sham, VPA + vehicle, and VPA + PGRN groups at PND35. The images were captured using a confocal microscope. Original magnification: 200×. Scale bar: 250 μm. Sample size of rats per group (*n*): *n* = 3. To minimize sampling bias, each brain tissue of rats was sectioned continuously. Subsequently, 3 slices with uniform thickness intervals were selected for further analysis. From each slice, 2–3 fields of view were chosen for detailed examination. Each dot in the graph corresponds to the outcome of an individual test conducted on a specific cell. Statistical analysis was performed using one‐way ANOVA followed by Sidak post hoc test. Data are presented as mean ± SEM (ns: no significant difference, ***p < 0.001).

To further explore whether PGRN regulates microglial activation, we performed immunofluorescence staining to examine the morphology and quantity of microglia in the hippocampus of the three groups of rats at PND35. ImageJ software was used to analyze the morphology of Iba‐1 positive cells in each group, including binary processing and noise reduction, outline extraction, skeleton analysis, and fractal dimension analysis, the results of which were labeled as binary, outline, skeleton, and hull and circle, respectively (Figure 3A). We applied principal component analysis (PCA) to evaluate eight indicators related to microglial morphology (Figure 3B). The analysis extracted the first three principal components: PC1, PC2, and PC3. These accounted for 38.58%, 20.93%, and 18.31% of the total variance, respectively, cumulatively contributing to 77.82% of the variance. Two biplots were utilized to provide a comprehensive display of both the loadings of the eight indicators and the PC scores of microglia from each group. The results distinctly indicated that microglia in the VPA + vehicle group exhibited a rounder cell body, shorter branches, and a more complex typing dimension. This suggests that the cells in this group were more akin to an amoeboid activation state. In contrast, microglial activation appeared to be inhibited following PGRN supplementation.

We also assessed the microglial cell counts in the hippocampus of the three groups (Figure 3C). The Iba1‐positive cell count was remarkably increased in the VPA + vehicle group compared to the Sham group, while it was significantly decreased in the VPA + PGRN group (*F*_2,64_ = 58.8, *p* < 0.001; post hoc, *p* < 0.001 for VPA vs. CON, *p* < 0.001 for VPA + PGRN vs. VPA + vehicle, Figure 3K). These findings provide additional evidence that the activated microglia can be inhibited by PGRN supplementation.

Based on the aforementioned results, PGRN can inhibit microglial activation and the NF‐κB pathway in the hippocampus of VPA‐induced ASD rats.

### 3.3 PGRN alleviated dendritic spine injury in the hippocampus of VPA‐induced ASD rats

**FIGURE 4:**
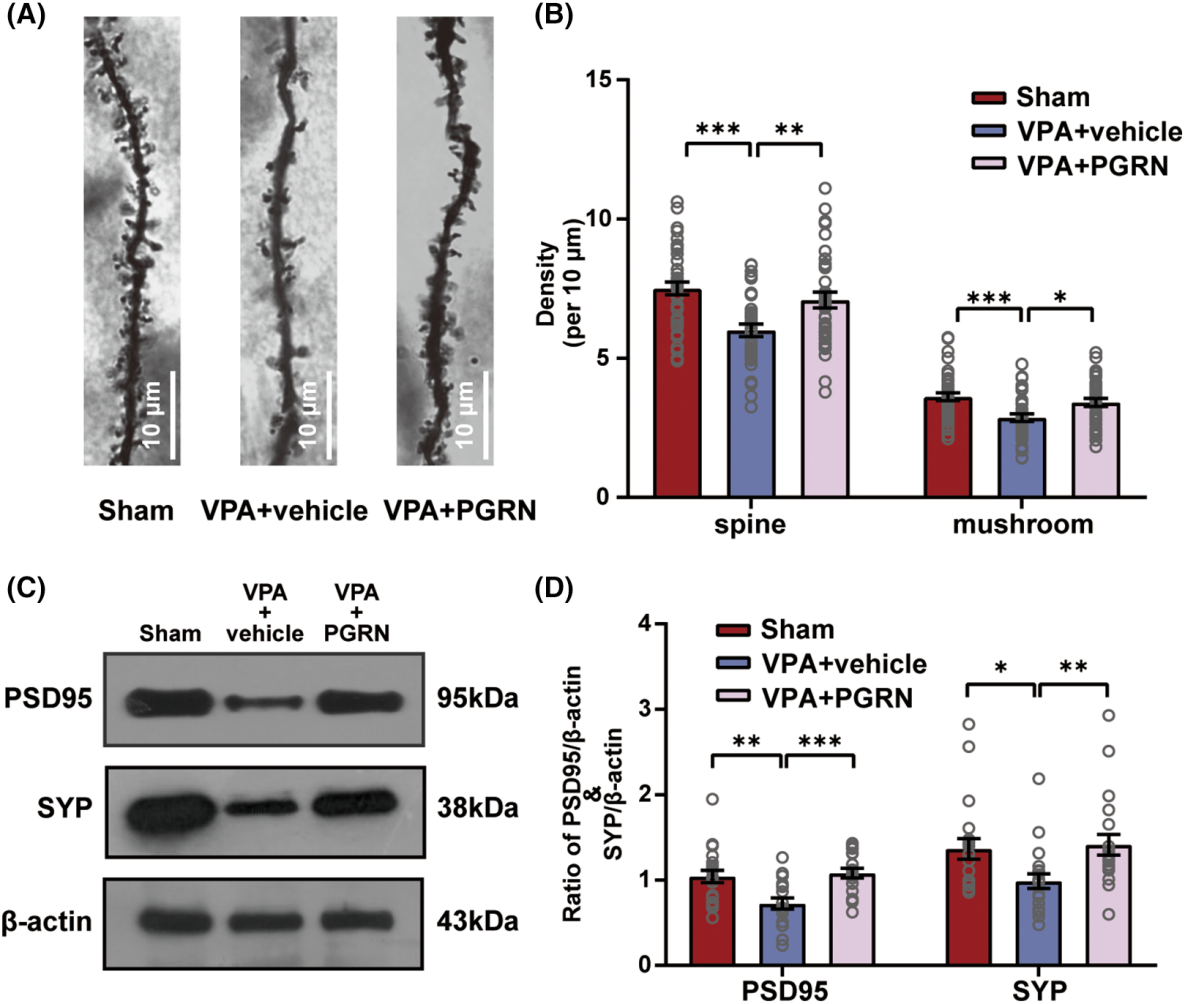
Impact of PGRN supplementation on dendritic spine injury in the hippocampus. (A) Representative images of Golgi‐Cox‐stained secondary dendritic spine morphology in the hippocampus of the Sham, VPA + vehicle, and VPA + PGRN groups at PND35. Original magnification: 1000×. Scale bar: 10 μm. (B) Quantification of total spine density and mushroom density per 10 μm. Sample size of rats per group (*n*): *n* = 3. To minimize sampling bias, each brain tissue of rats was sectioned continuously. Subsequently, 3 slices with uniform thickness intervals were selected for further analysis. From each slice, 5 dendrites were chosen for detailed examination. Each dot in the graph corresponds to the outcome of an individual test conducted on a specific dendrite. (C and D) Western blot and quantification analysis of the protein levels of PSD95 and SYP in the hippocampus of the Sham, VPA + vehicle, and VPA + PGRN groups at PND35. Sample size (*n*): *n* = 5/group. The analysis was repeated five times. Statistical analysis was performed using one‐way ANOVA followed by Sidak post hoc test. Data are presented as mean ± SEM (ns: no significant difference, *p < 0.05, **p < 0.01, ***p < 0.001).

To explore whether PGRN can influence the synaptic development that occurred following the inhibition of the NF‐kB pathway and microglial activation, we employed Golgi‐Cox staining to examine the density and morphology of secondary dendritic spines of the hippocampal neurons in the three groups of rats at PND35 (Figure 4A). The total density of dendritic spines and the density of mushroom‐shaped dendritic spines in the hippocampal neurons exhibited a significant decrease in the VPA + vehicle group, however, both these parameters were significantly increased in the VPA + PGRN group (total dendritic spines: *F*_2,109_ = 9.393, *p* < 0.001; post hoc, *p* < 0.001 for VPA + vehicle vs. Sham, *p* = 0.006 for VPA + PGRN vs. VPA + vehicle; mushroom‐shaped dendritic spines: *F*_2,109_ = 7.854, *p* < 0.001; post hoc, *p* < 0.001 for VPA + vehicle vs. Sham, *p* = 0.01 for VPA + PGRN vs. VPA + vehicle, Figure 4B). Furthermore, we conducted western blotting to assess the expression of synaptic development‐related proteins, PSD95 and SYP, in the hippocampus (Figure 4C). In the VPA + vehicle group, the decreased levels of PSD95 and SYP partially rebounded following PGRN supplementation (PSD95: *F*_2,57_ = 8.887, *p* < 0.001; post hoc, *p* = 0.002 for VPA + vehicle vs. Sham, *p* < 0.001 for VPA + PGRN vs. VPA + vehicle; SYP: Kruskal‐Wallis statistic = 12.9, *p* = 0.002; post hoc, *p* = 0.01 for VPA + vehicle vs. Sham, *p* = 0.002 for VPA + PGRN vs. VPA + vehicle, Figure 4D). This observation was in line with the findings from the Golgi–Cox staining, further substantiating that PGRN supplementation effectively alleviated the dendritic spine injury in VPA‐induced ASD rats.

### 3.4 Suppression of the elevated sortilin level upregulated the PGRN expression in the hippocampus of VPA‐induced ASD rats

**FIGURE 5:**
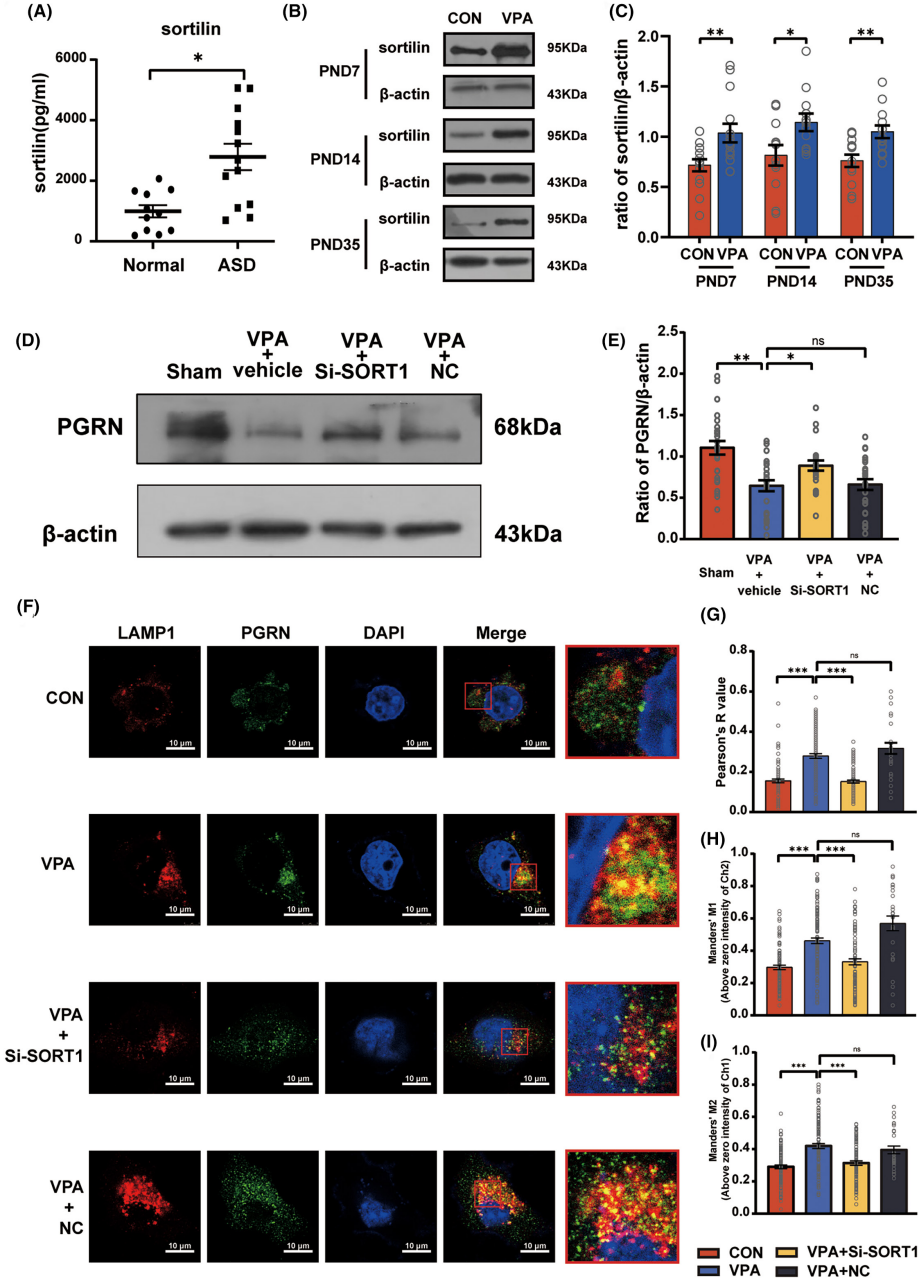
Evaluation of sortilin level in ASD and the impact of *SORT1* knockdown on PGRN. (A) ELISA showed serum sortilin levels in normal children and children with ASD. Sample size (*n*): *n* = 11 (normal) and *n* = 13 (ASD), using unpaired *t* test for statistical analysis. (B and C) Western blot and quantification analysis of the sortilin protein level in the hippocampus of the CON and VPA groups at PND7, PND14, and PND35. Sample size (*n*): *n* = 4/group. The analysis was repeated four times, using an unpaired *t* test for statistical analysis. (D and E) Western blot and quantification analysis of the PGRN protein level in the hippocampus of the Sham, VPA + vehicle, VPA + Si‐SORT1, and VPA + NC at PND35. Sample size (*n*): *n* = 5/group. The analysis was repeated five times, using one‐way ANOVA followed by Sidak post hoc test for statistical analysis. (F) Representative images demonstrating the co‐localization of PGRN and LAMP1 in the BV2 microglial cells of the CON, VPA, VPA + Si‐SORT1, and VPA + NC groups. The images were obtained using a confocal microscope. Red box: Enlarged image of selected cells demonstrating the details. Original magnification: 2000×. Scale bar: 10 μm. Quantification analysis of Pearson's R value (G), Manderson's M1 (H), and Manderson's M2 (I) were measured using Coloc2 analysis in ImageJ software. Sample size of petri dishes per group (*n*): *n* = 3/group. For each sample, 10 fields of vision encompassing 30 cells were selected and the analysis was repeated three times. Data are presented as mean ± SEM (ns: no significant difference, *p < 0.05, **p < 0.01, ***p < 0.001).

In the present study, ELISA results showed higher serum sortilin levels in ASD children compared to normal children (*t* = 3.534, *df* = 22, *p* = 0.0019 for normal vs. ASD, Figure 5A). Furthermore, we found a significant increase in the sortilin expression level in the hippocampus of the VPA group at PND7, PND14, and PND35 (PND7:*t* = 2.455, *df* = 20, *p* = 0.0234 for VPA vs. CON; PND14: *t* = 2.172, df = 16, *p* = 0.0452 for VPA vs. CON; PND35:*t* = 3.192, *df* = 18, *p* = 0.0051 for VPA vs. CON, Figure 5C).

Next, inhibition of the sortilin expression led to a significant reduction in the PGRN expression in the VPA + vehicle group and significantly increased the PGRN expression in the VPA + Si‐SORT1 group (*F*_3,96_ = 9.825, *p* < 0.001; post hoc, *p* < 0.001 for VPA + vehicle vs. Sham, *p* = 0.04 for VPA + Si‐SORT1 vs. VPA + vehicle; *p* = 0.88 for VPA + NC vs. VPA + vehicle, Figure 5E).

To accurately determine the impact of sortilin on PGRN, we cultured the BV2 microglia cells in vitro and then treated them with VPA (2.5 mmol/L) to simulate the effects of VPA on the microglia in the nervous system. Immunofluorescence staining was performed to visualize the localization of anti‐PGRN and anti‐LAMP1 (Figure 5F), followed by co‐localization analysis (Figure 5G–I). The VPA + Si‐SORT1 group displayed a significant reduction in the Manders' M1, Manders' M2, and Pearson's R values (Pearson's R value: Kruskal‐Wallis statistic = 96.56, *p* < 0.001; post hoc, *p* < 0.001 for VPA vs. CON, *p* < 0.001 for VPA + Si‐SORT1 vs. VPA; *p* > 0.99 for VPA + NC vs. VPA; Manders' M1: Kruskal–Wallis statistic = 61.28, *p* < 0.001; post hoc, *p* < 0.001 for VPA vs. CON, *p* < 0.001 for VPA + Si‐SORT1 vs. VPA; *p* = 0.25 for VPA + NC vs. VPA; Manders' M2: Kruskal‐Wallis statistic = 42.55, *p* < 0.001; post hoc, *p* < 0.001 for VPA vs. CON, *p* < 0.001 for VPA + Si‐SORT1 vs. VPA; *p* > 0.99 for VPA + NC vs. VPA). These findings suggest that inhibition of the sortilin expression reduced the PGRN degradation by lysosomes, leading to an increase in the PGRN protein levels.

### 3.5 Suppression of sortilin expression inhibited the microglial activation and inflammation in the hippocampus of VPA‐induced ASD rats

**FIGURE 6:**
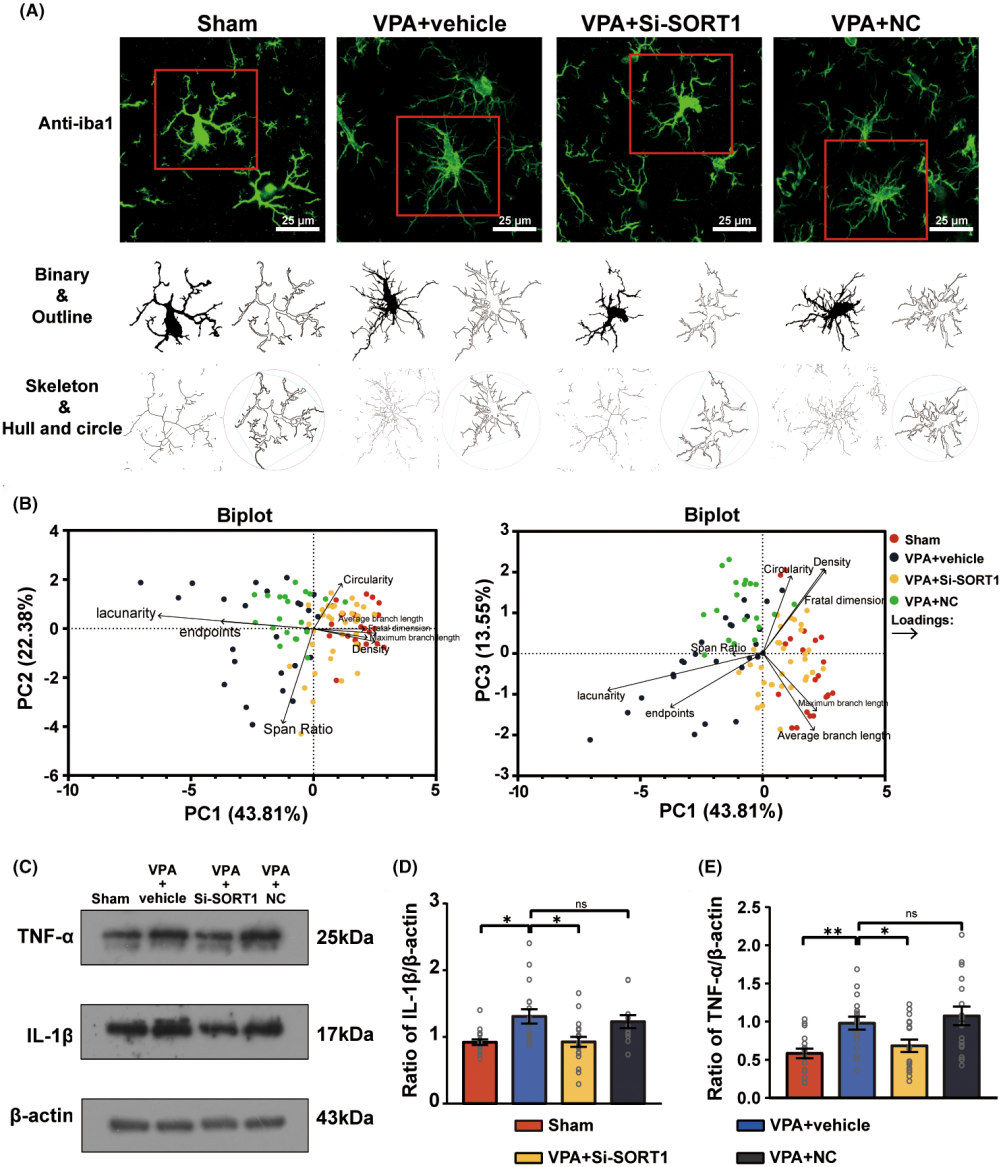
Impact of *SORT1* knockdown on the microglial morphology and inflammation in the hippocampus. (A) Representative images of Iba1‐stained microglial morphology in the hippocampus of the CON, VPA, VPA + Si‐SORT1, and VPA + NC groups at PND35. The images were captured using a confocal microscope. Original magnification: 630×. Scale bar: 25 μm. Cropped and analyzed photomicrographs (below) demonstrating additional details. All analyses were performed on full‐sized photomicrographs. (B) Principal component analysis (PCA) of the morphological changes of microglia in the hippocampus of the Sham, VPA + vehicle, VPA + Si‐SORT1, and VPA + PGRN group. The figure displays a biplot for PCA, analyzing eight morphological change indicators: Endpoints, average branch length, maximum branch length, density, span ratio, circularity, lacunarity, and fractal dimension. Percentages of variation for morphology of microglia are shown for the first three principal components (PC1, PC2, and PC3). In the biplot, each point represents the principal component (PC) score of a microglia, and arrows represent loadings. The length of each arrow indicates the relative contribution of each indicator to the PCA, and the angles between arrows illustrate their correlations. Sample size of rats per group (*n*): *n* = 3. To minimize sampling bias, each brain tissue of rats was sectioned continuously. Subsequently, 3 slices with uniform thickness intervals were selected for further analysis. From each slice, 2–3 fields of view were chosen for detailed examination. Sample size of microglia per group (*n*): *n* = 20 (Sham), *n* = 25 (VPA + vehicle), *n* = 34 (VPA + Si‐SORT1), *n* = 20 (VPA + Si‐NC). Each dot in the graph corresponds to the outcome of an individual test conducted on a specific cell. (C–E) Western blot and quantification analysis of the protein levels of TNF‐α and IL‐1β in the hippocampus of the CON, VPA, VPA + Si‐SORT1, and VPA + NC groups at PND35. Sample size (*n*): *n* = 5/group. The analysis was repeated three times, using one‐way ANOVA followed by Sidak post hoc test for statistical analysis. Data are presented as mean ± SEM (ns: No significant difference, *p < 0.05, **p < 0.01).

To determine whether sortilin inhibition promoted microglial activation and inflammation, we continued to use immunofluorescence staining to conduct the morphological analysis of microglia in the hippocampus (Figure 6A), and applied PCA to evaluate microglial morphology (Figure 6B). The analysis extracted the first three principal components: PC1, PC2, and PC3, which accounted for 43.81%, 22.38%, and 13.55% of the total variance, respectively, cumulatively contributing to 79.74% of the variance. The biplots distinctly indicated that after *SORT1* knockdown, microglial activation appeared to be inhibited.

Moreover, the western blot analysis demonstrated a notable decrease in both IL‐1β and TNF‐α levels following *SORT1* knockdown (Figure 6C–E) (IL‐1β: Kruskal‐Wallis statistic = 16.66, *p* < 0.001; post hoc, *p* = 0.004 for VPA + vehicle vs. Sham, *p* = 0.01 for VPA + Si‐SORT1 vs. VPA + vehicle; *p* > 0.99 for VPA + NC vs. VPA + vehicle; TNF‐α: *F*_3,64_ = 2.233, *p* = 0.09; post hoc, *p* = 0.009 for VPA + vehicle vs. Sham, *p* = 0.05 for VPA + Si‐SORT1 vs. VPA + vehicle; *p* = 0.46 for VPA + NC vs. VPA + vehicle).

These results indicate that sortilin inhibition can inhibit microglial activation and reduce inflammation in VPA‐induced ASD rats.

### 3.6 Suppression of sortilin expression alleviated autism‐like behavior in VPA‐induced ASD rats

**FIGURE 7:**
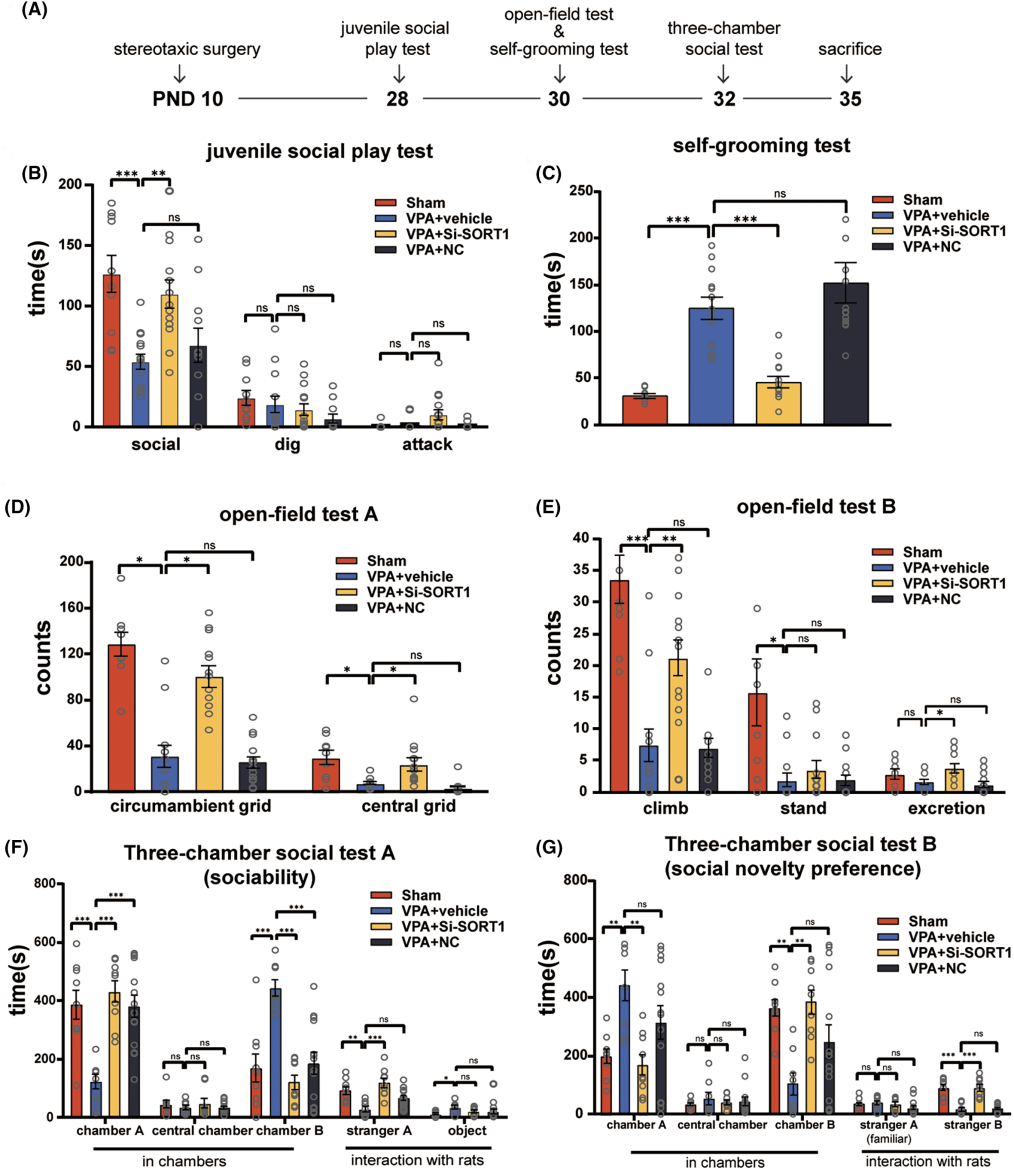
PGRN level in the hippocampus of VPA‐induced ASD rats and the impact of PGRN supplementation on autism‐like behaviors. (A) Timeline of the stereotaxic surgery, autism‐related behavioral tests, and brain harvesting. (B) Results from the juvenile social play test showing the social time, digging time, and attack time. (C) Results from the self‐grooming test showing the grooming time. (D and E) Open‐field test showing the central grid, circumambient grid, climb, stand, and excretion. (F and G) Results from the three‐chamber social test showing the time spent in the central, A, and B chambers, and interaction with strangers A and B. Sample size (*n*): *n* = 9 (Sham), *n* = 8 (VPA + vehicle), *n* = 9 (VPA + Si‐SORT1), and *n* = 14 (VPA + NC). Data are presented as mean ± SEM. One‐way ANOVA and the Sidak post hoc or Kruskal–Wallis tests were used for multiple comparisons (ns: no significant difference, *p < 0.05, **p < 0.01, ***p < 0.001).

To examine whether sortilin suppression alleviates autism‐like behaviors, we used the same behavioral testing protocol as previously described to assess the autism‐like behaviors in the four groups of rats during PND28‐32. The timeline of this evaluation is depicted in the corresponding Figure 7A.

The juvenile social play test (Figure 7B) showed a notable increase in social time with *SORT1* knockdown (*F*_3,44_ = 8.728, *p* < 0.001; post hoc, *p* < 0.001 for VPA + vehicle vs. Sham, *p* = 0.002 for VPA + Si‐SORT1 vs. VPA + vehicle, *p* = 0.74 for VPA + NC vs. VPA + vehicle). The Self‐Grooming test (Figure 7C) demonstrated a significant reduction in the grooming duration with *SORT1* knockdown (Kruskal‐Wallis statistic = 34.86, *p* < 0.001; post hoc, *p* < 0.001 for VPA + vehicle vs. Sham, *p* < 0.001 for VPA + Si‐SORT1 vs. VPA + vehicle, *p* > 0.99 for VPA + NC vs. VPA + vehicle). Furthermore, in the open‐field test (Figure 7D,E), the VPA + Si‐SORT1 group exhibited an increase in the number of climbing events, circumambient grid crossings, and central grid crossings (climb: Kruskal‐Wallis statistic = 25.93, *p* < 0.001; post hoc, *p* < 0.001 for VPA + vehicle vs. Sham, *p* = 0.008 for VPA + Si‐SORT1 vs. VPA + vehicle, *p* > 0.99 for VPA + NC vs. VPA + vehicle; circumambient grids: *F*_3,45_ = 33.01, *p* < 0.001; post hoc, *p* < 0.001 for VPA + vehicle vs. Sham, *p* < 0.001 for VPA + Si‐SORT1 vs. VPA + vehicle, *p* = 0.96 for VPA + NC vs. VPA + vehicle; central grids: Kruskal‐Wallis statistic = 28.1, *p* < 0.001; post hoc, *p* = 0.02 for VPA + vehicle vs.Sham, *p* = 0.04 for VPA + Si‐SORT1 vs. VPA + vehicle, *p* = 0.18 for VPA + NC vs. VPA + vehicle).

During the first stage of the three‐chamber social test, the VPA + Si‐SORT1 group exhibited a marked increase in the time spent in chamber A and interaction with stranger A, while the time spent in chamber B was significantly decreased (chamber A: *F*_3,36_ = 10.25, *p* < 0.001; post hoc, *p* < 0.001 for VPA + vehicle vs. Sham, *p* < 0.001 for VPA + Si‐SORT1 vs. VPA + vehicle, *p* < 0.001 for VPA + NC vs. VPA + vehicle; stranger A: *F*_3,36_ = 9.724, *p* < 0.001; post hoc, *p* = 0.002 for VPA + vehicle vs. Sham, *p* < 0.001 for VPA + Si‐SORT1 vs. VPA + vehicle, *p* = 0.08 for VPA + NC vs. VPA + vehicle; chamber B: *F*_3,36_ = 12.38, *p* < 0.001; post hoc, *p* < 0.001 for VPA + vehicle vs. Sham, *p* < 0.001 for VPA + Si‐SORT1 vs. VPA + vehicle, *p* < 0.001 for VPA + NC vs. VPA + vehicle, Figure 7F). During the second stage, the time spent in chamber A was notably decreased, while the time spent in chamber B and the duration of interaction with stranger B were significantly increased (chamber A: *F*_3,36_ = 5.296, *p* = 0.004; post hoc, *p* = 0.009 for VPA + vehicle vs. Sham, *p* = 0.003 for VPA + Si‐SORT1 vs. VPA + vehicle, *p* = 0.2 for VPA + NC vs. VPA + vehicle; chamber B: *F*_3,36_ = 5.752, *p* = 0.003; post hoc, *p* = 0.005 for VPA + vehicle vs. Sham, *p* = 0.002 for VPA + Si‐SORT1 vs. VPA + vehicle, *p* = 0.13 for VPA + NC vs. VPA + vehicle; stranger B: Kruskal‐Wallis statistic = 28.5, *p* < 0.001; post hoc, *p* < 0.001 for VPA + vehicle vs. Sham, *p* < 0.001 for VPA + Si‐SORT1 vs. VPA + vehicle, *p* > 0.99 for VPA + NC vs. VPA + vehicle, Figure 7G).

Collectively, these results suggest that sortilin inhibition can reduce repetitive and rigid behavior, enhance sociability, and mitigate impaired social preference in VPA‐induced ASD rats.

We also monitored the body weight, general appearance, porphyrin staining, gait and posture, appetite, and wound condition of the animals pre‐surgery and on postoperative days 1, 3, and 7 to exclude any traumatic damage to rats during the Lentivirus injection process. We found that the surgery did not cause significant trauma in the sham group, VPA + vehicle group, VPA + si‐*SORT1* group, and VPA + NC group animals (see Table S2). Therefore, we believe that the behavioral changes observed are mainly influenced by the knockdown of *SORT1*, rather than by the surgery itself.

## 4 DISCUSSION

ASD is a group of neurodevelopmental disorders. Some studies have demonstrated that microglial activation and inflammation are recognized factors involved in ASD pathogenesis.^33^, ^34^ In line with this, we observed increased serum TNF‐α in children with ASD and in the hippocampus of VPA‐induced ASD rats (Figure 2). The underlying mechanism, however, remains a topic of debate.

Our investigation identified a reduction in PGRN expression in the serum of children with autism and in the hippocampus of VPA‐induced ASD rats (Figure 1). PGRN, a protein secreted by microglia and neurons, has a variety of biological functions under different conditions. Its deficiency has been linked to persistent microgliosis and increased neuronal loss in brain injuries,^35^, ^36^ and can cause frontotemporal dementia, a cognitive disorder.^37^ Additionally, PGRN deficiency results in excessive NF‐κB activation in microglia, indicating its anti‐inflammatory effects.^38^ However, contrary findings exist. For instance, a study revealed that single ICV administration of rPGRN (4 μg/mice) didn't provide the anticipated protective effects in the acute phase of murine TBI, but rather exacerbated blood–brain barrier disruption.^39^ We found a reduction of PGRN in the hippocampus of VPA‐induced ASD rats. Interestingly, our results showed that rPGRN supplemention significantly reduced autism‐like behavior, such as stereotyped behavior and social impairment (Figure 1), and downregulated the NF‐κB pathway, inhibiting microglial activation in the hippocampus of VPA‐induced ASD rats (Figures 2 and 3), aligning with Fu et al.'s findings.^40^ Certainly, the activation of the NF‐κB pathway may also induce microglial activation simultaneously.^11^ These discrepancies could be attributed to variations in the timing and dosage of rPGRN injections, the chosen time points for evaluating test subjects, and the specific disease backgrounds of the model animals.

ASD is characterized by abnormal development of neuronal dendrites.^41^, ^42^ Studies have identified that NF‐κB activation can lead to neuron and dendrite defects, including spine loss and synaptic dysfunction.^43^, ^44^, ^45^, ^46^ Microglia are key contributors to neuronal development and participate in synaptic pruning during critical stages.^47^, ^48^ Microglial activation and synaptic engulfment contribute to the loss of synapses and reduction in dendritic spines in the hippocampus, leading to neurocognitive dysfunction and the development of anxiety‐like behaviors.^49^ Importantly, PGRN supplementation in our study increased the density of secondary dendritic spines and mature dendrites in hippocampal neurons, and enhanced PSD95 and SYP expression levels (Figure 4). Based on these findings, we propose that PGRN alleviates ASD‐like symptoms through direct anti‐inflammatory effects on the nervous system and inhibits excessive microglial activation, thereby alleviating abnormal synapse development.

Microglia are the primary source of PGRN in the brain. Some research indicates an increase in PGRN levels following microglia activation,^50^ which is not consistent with our observations. This discrepancy may be attributed to the degradation process of PGRN, which also influences its expression levels. Therefore, in our study, the overall PGRN levels may reflect not just its production by activated microglia but also the rate and efficiency of its degradation, suggesting a more complex regulatory mechanism of PGRN in neurobiological contexts.

In vivo, PGRN is governed by endocytosis with the help of sortilin or through alternative PSAP (prosaposin)‐dependent pathway with involvement of mannose 6‐phosphate receptor (M6PR) and low density lipoprotein receptor‐related protein 1 (LRP1).^51^, ^52^ This can explain why some functions of PGRN are independent of sortilin.^53^ Even though there are different modes of transport, sortilin has a predominant role in regulating PGRN expression.^54^ Extracellular PGRN levels can be reduced by sortilin.^55^ Sortilin is a direct high‐affinity receptor for PGRN, binding to the C‐terminal tail of PGRN and trafficking it into the lysosome for degradation.^24^, ^25^ In the nervous system, sortilin's role in regulating PGRN is controversial. Valentina Gumina et al. found that in murine models of FTLD, targeting the progranulin‐sortilin axis increases extracellular PGRN levels, but this was not replicated in human FTLD neuronal cell models.^56^ Therefore, the relationship between sortilin and PGRN in ASD rats needs to be observed.

In our study, we found significantly elevated serum levels of sortilin in children with ASD and the hippocampus in ASD model rats, accompanied by a decrease in serum levels of PGRN in children with ASD (Figure 1). Suppression of these elevated sortilin levels upregulated PGRN expression in the hippocampus (Figure 5). At the same time, we noted a significant overlap between LAMP1 and PGRN in the VPA group, utilizing SORT1 knockdown, this overlap was alleviated. Despite intracellular PGRN can initially localized to lysosomes as a chaperone for lysosomal enzymes,^57^ full‐length PGRN is unstable in lysosomes, with endocytosed PGRN rapidly processed into mature, stable short GRNs finally.^58^ Hence, we speculated that the PGRN deficiency might be associated with the elevation of sortilin in the ASD model. Furthermore, we observed that sortilin suppression mitigated inflammation in the hippocampus of VPA‐induced rats and alleviated autism‐like behavior in these animals (Figures 6 and 7). We infer that elevated sortilin levels in ASD rat brains may play a role in ASD by affecting PGRN expression.

In conclusion, we found low PGRN expression levels in the hippocampus of VPA‐induced ASD rats, which is associated with high levels of sortilin. PGRN supplementation or sortilin inhibition could attenuate synaptic injury and alleviated autism‐like behaviors via anti‐microglial activation and anti‐inflammatory effects. These insights underscore the potential of targeted therapies, paving the way for the development of novel therapeutic interventions for ASD.

However, we acknowledge the limited direct therapeutic applicability of stereotactic injections in clinical settings for autism treatment. Strategies to inhibit sortilin or elevate PGRN levels in a safe and non‐invasive manner^59^, ^60^, ^61^, ^62^ are ongoing and offer further hope for preventing or reversing behavioral deficits in ASD in the future.

## AUTHOR CONTRIBUTIONS

**Ailing Liao**: Investigation, Validation, Formal analysis, Writing‐Original Draft, Visualization. **Wenxia Zheng:** Data collection and Curation. **Shali Wang, Nashi Wang, Yingbo Li, Di Chen:** Resources, Funding acquisition and Supervision. **Yan Wang**: Conceptualization, Methodology, Writing‐Reviewing and Editing.

## FUNDING INFORMATION

This work was supported by the Chongqing Children's Aid Foundation (Life and Social Interaction Enhancement Program for Children with Autism); Science and Technology Research Program of Chongqing Education Commission of China. (Grant No. KJQN202300437); Natural Science Foundation of Chongqing, China. (No. cstc2021jcyj‐msxmX0065).

## CONFLICT OF INTEREST STATEMENT

The authors declare that there are no conflicts of interest.

## Supporting information


Table S1.

Table S2.

## Data Availability

The data that support the findings of this study are available on request from the corresponding author. The data are not publicly available due to privacy or ethical restrictions.
