# Supplementary material for: Sortilin is associated with progranulin deficiency and autism‐like behaviors in valproic acid‐induced autism rats
Source: CNS Neurosci Ther. 2024 Sep 1;30(9):e70015. doi: 10.1111/cns.70015 (PMC11366450; doi:10.1111/cns.70015)
Supplement: Supplementary file 1 — Table S1. [file CNS-30-e70015-s001.pdf]

Supplementary Table 1: Postoperative monitoring data of rPGRN injection

Surgery Type	Group	Baseline Body Weight （g）	Postoperative Time Point	General appearance （score）	Porphyrin staining （score）	Gait and posture （score）	Body weight （g）	Body weight loss from baseline（%）	Body weight loss from baseline（score）	Appetite （score）	Wound condition （score）	Total score
**First Surgury**	**Sham**	18.84±0.96	**Day1**	0.09±0.03	0.00	0.01±0.03	17.98±0.91	4.52%±1.65%	0.04±0.05	0.00	0.00	0.14±0.08
			**Day3**	0.00	0.00	0.01±0.03	19.02±0.99	0.97%±2.79%	0.00	0.00	0.00	0.01±0.03
			**Day7**	0.00	0.00	0.00	29.58±1.80	-57.27%±9.24%	0.00	0.00	0.00	0.00
	**VPA+vehicle**	18.55±0.76	**Day1**	0.10	0.01±0.03	0.00	18.03±0.65	2.54%±1.17%	0.00	0.00	0.00	0.11±0.03
			**Day3**	0.00	0.01±0.03	0.00	19.13±0.75	-3.12%±1.55%	0.00	0.00	0.00	0.01±0.03
			**Day7**	0.00	0.00	0.00	30.13±1.10	-62.49%±5.73%	0.00	0.00	0.00	0.00
	**VPA+PGRN**	20.21±2.05	**Day1**	0.1±0.02	0±0.02	0.01±0.03	19.62±2.14	3.00%±1.71%	0.01±0.04	0.00	0.00	0.12±0.05
			**Day3**	0±0.02	0.00	0.01±0.03	20.87±2.04	-3.29%±1.46%	0.00	0.00	0.00	0.01±0.04
			**Day7**	0.00	0.00	0.00	31.35±2.06	-55.63%±6.60%	0.00	0.00	0.00	0.00
**Second Surgury**	**Sham**	29.62±1.79	**Day1**	0.09±0.03	0.00	0.01±0.03	29.09±1.74	1.75%±1.15%	0.00	0.00	0.00	0.1±0.04
			**Day3**	0.02±0.04	0.00	0.01±0.03	29.22±1.64	1.27%±2.38%	0.00	0.00	0.00	0.02±0.04
			**Day7**	0.00	0.00	0.00	52.03±3.98	-74.32%±20.58%	0.00	0.00	0.00	0.00
	**VPA+vehicle**	29.99±1.10	**Day1**	0.10	0.01±0.03	0.00	28.77±0.76	3.46%±1.29%	0.02±0.04	0.00	0.00	0.13±0.06
			**Day3**	0.02±0.04	0.03±0.06	0.00	30.07±0.90	-0.35%±4.31%	0.00	0.00	0.00	0.04±0.07
			**Day7**	0.00	0.00	0.00	50.08±1.18	-67.13%±5.61%	0.00	0.00	0.00	0.00
	**VPA+PGRN**	31.35±2.05	**Day1**	0.09±0.03	0±0.02	0.01±0.03	30.08±1.91	4.02%±1.38%	0.03±0.05	0.00	0.00	0.13±0.05
			**Day3**	0.04±0.05	0±0.02	0.01±0.03	31.86±2.22	1.74%±5.36%	0.00	0.00	0.00	0.05±0.06
			**Day7**	0.00	0.00	0.00	51.20±2.00	-63.61%±4.32%	0.00	0.00	0.00	0.00
