# Supplementary material for: Sortilin is associated with progranulin deficiency and autism‐like behaviors in valproic acid‐induced autism rats
Source: CNS Neurosci Ther. 2024 Sep 1;30(9):e70015. doi: 10.1111/cns.70015 (PMC11366450; doi:10.1111/cns.70015)
Supplement: Supplementary file 2 — Table S2. [file CNS-30-e70015-s002.pdf]

Supplementary Table 2: Postoperative monitoring data of Lentivirus injection

Group	Baseline Body Weight（g）	Postoperative Time Point	General appearance（score）	Porphyrin staining（score）	Gait and posture（score）	Body weight（g）	Body weight loss from baseline（%）	Body weight loss from baseline（score）	Appetite（score）	Wound condition（score）	Total score
**Sham**	19.35±1.21	**Day1**	0.10	0.01±0.03	0.01±0.03	18.3±1.25	5.44%±1.92%	0.05±0.05	0.00	0.00	0.17±0.07
		**Day3**	0.05±0.05	0.00	0.01±0.03	19.25±1.43	0.57%±1.89%	0.00	0.00	0.00	0.06±0.07
		**Day7**	0.00	0.00	0.00	28.79±1.02	-49.17%±8.47%	0.00	0.00	0.00	0.00
**VPA+vehicle**	20.18±0.74	**Day1**	0.10	0.01±0.04	0.01±0.04	19.26±0.74	4.50%±2.41%	0.03±0.05	0.00	0.00	0.15±0.05
		**Day3**	0.04±0.05	0.01±0.04	0.00	20.50±1.13	-1.69%±5.97%	0.00	0.00	0.00	0.05±0.05
		**Day7**	0.00	0.00	0.00	29.85±1.79	-47.90%±5.19%	0.00	0.00	0.00	0.00
**VPA+si-*SORT1***	20.19±0.88	**Day1**	0.10	0.01±0.03	0.02±0.04	19.03±1.08	5.75%±2.18%	0.07±0.05	0.00	0.00	0.2±0.09
		**Day3**	0.02±0.04	0.01±0.03	0.01±0.03	19.78±1.02	2.05%±1.60%	0.00	0.00	0.00	0.04±0.07
		**Day7**	0.00	0.00	0.00	28.98±0.73	-43.65%±3.50%	0.00	0.00	0.00	0.00
**VPA+NC**	19.30±1.05	**Day1**	0.10	0.01±0.04	0.00	18.26±0.91	5.36%±2.16%	0.06±0.05	0.00	0.00	0.17±0.06
		**Day3**	0.07±0.05	0.01±0.03	0.00	19.00±0.96	1.51%±2.21%	0.00	0.00	0.00	0.08±0.06
		**Day7**	0.00	0.00	0.00	29.36±2.45	-52.24%±11.21%	0.00	0.00	0.00	0.00
